# Supplementary material for: Absence of Microglial Activation and Maintained Hippocampal Neurogenesis in a Transgenic Mouse Model of Crohn’s Disease
Source: Cells. 2025 Jun 4;14(11):841. doi: 10.3390/cells14110841 (PMC12155506; doi:10.3390/cells14110841)
Supplement: Supplementary file 1 [file cells-14-00841-s001.zip › cells-3584380-supplementary.pdf]

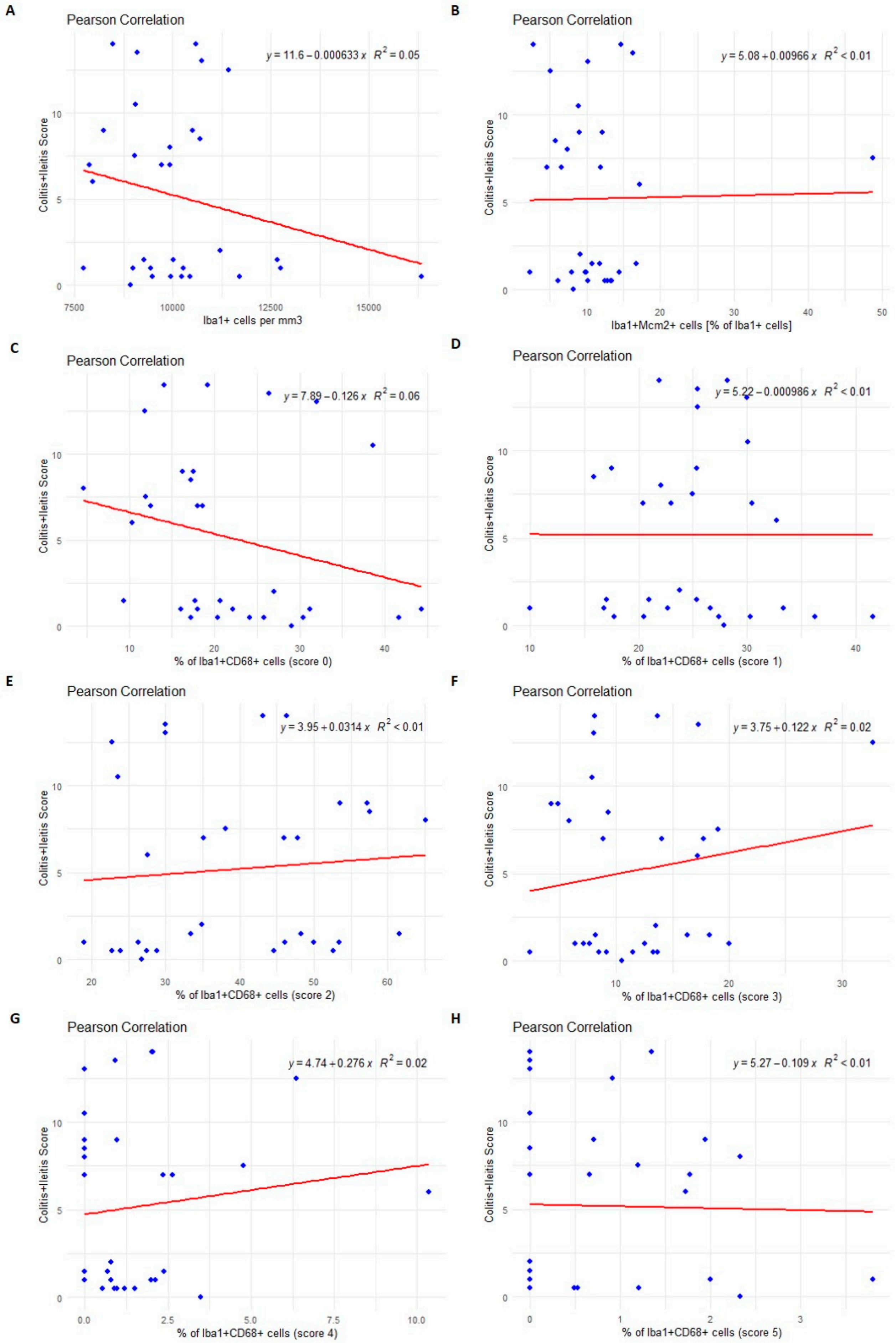
A
Pearson Correlation
$y = 11.6 - 0.000633\,x$ $R^2 = 0.05$
Colitis+Ileitis Score
Iba1+ cells per mm3
B
Pearson Correlation
$y = 5.08 + 0.00966\,x$ $R^2 < 0.01$
Colitis+Ileitis Score
Iba1+Mcm2+ cells [% of Iba1+ cells]
C
Pearson Correlation
$y = 7.89 - 0.126\,x$ $R^2 = 0.06$
Colitis+Ileitis Score
% of Iba1+CD68+ cells (score 0)
D
Pearson Correlation
$y = 5.22 - 0.000986\,x$ $R^2 < 0.01$
Colitis+Ileitis Score
% of Iba1+CD68+ cells (score 1)
E
Pearson Correlation
$y = 3.95 + 0.0314\,x$ $R^2 < 0.01$
Colitis+Ileitis Score
% of Iba1+CD68+ cells (score 2)
F
Pearson Correlation
$y = 3.75 + 0.122\,x$ $R^2 = 0.02$
Colitis+Ileitis Score
% of Iba1+CD68+ cells (score 3)
G
Pearson Correlation
$y = 4.74 + 0.276\,x$ $R^2 = 0.02$
Colitis+Ileitis Score
% of Iba1+CD68+ cells (score 4)
H
Pearson Correlation
$y = 5.27 - 0.109\,x$ $R^2 < 0.01$
Colitis+Ileitis Score
% of Iba1+CD68+ cells (score 5)

Supplementary Figure S1. Correlation of microglial parameters with ileocolitis severity. Pearson correlation analysis revealed no correlation between Colitis+Ileitis score and (A) Iba1$^+$ cell density in the DG (R^2 = *-0.2200*), (B) percentage of Iba1$^+$Mcm2$^+$ cells of all Iba1$^+$ cells in the DG (R^2 = *0.0156*), and (C-H) microglial activation scores 0-5 (R^2 = *-0.2419*, R^2 = *-0.0013*, R^2 = *0.0845*, R^2 = *0.1517*, R^2 = *0.1230*, R^2 = *-0.0218*, respectively). Regression line equations and R^2 values are indicated in the respective plot.
